# P-selectin is a host receptor for *Plasmodium* MSP7 ligands

**DOI:** 10.1186/s12936-015-0750-z

**Published:** 2015-06-05

**Authors:** Abigail J Perrin, S Josefin Bartholdson, Gavin J Wright

**Affiliations:** Cell Surface Signalling Laboratory and Malaria Programme, Wellcome Trust Sanger Institute, Hinxton, Cambridge, CB10 1SA UK

**Keywords:** MSP7, P-selectin, Immune evasion, *Plasmodium falciparum*, Surface plasmon resonance, MSP1, MSRPs, Sialyl-Lewis-X

## Abstract

**Background:**

*Plasmodium* parasites typically elicit a non-sterile but protective immune response in human host populations, suggesting that the parasites actively modulate normal immunological mechanisms. P-selectin is a cell surface receptor expressed in mammals, that is a known component of the inflammatory response against pathogens and has been previously identified as a host factor that influences malaria-associated pathology both in human patients and rodent infection models.

**Methods:**

To better understand the molecular mechanisms underlying the involvement of P-selectin in the pathogenesis of malaria, a systematic extracellular protein interaction screen was used to identify *Plasmodium falciparum* merozoite surface protein 7 (MSP7) as a binding partner of human P-selectin. This interaction, and those occurring between P-selectin and *Plasmodium* MSP7 homologues, was characterized biochemically.

**Results:**

*Plasmodium falciparum* MSP7 and P-selectin were shown to bind each other directly *via* the N-terminus of PfMSP7 and the P-selectin C-type lectin and EGF-like domains. Orthologous proteins in the murine parasite *Plasmodium berghei* (PbMSRP1 and PbMSRP2) and mouse P-selectin also interacted. Finally, P-selectin, when complexed with MSP7, could no longer bind to its endogenous carbohydrate ligand, Sialyl-Lewis^X^.

**Conclusions:**

Novel interactions were identified between *Plasmodium* MSP7 protein family members and host P-selectin receptors. Since PfMSP7 could prevent interactions between P-selectin and its leukocyte ligands, these results provide a possible mechanism for the known immunomodulatory effects of both MSP7 and P-selectin in malaria infection models.

**Electronic supplementary material:**

The online version of this article (doi:10.1186/s12936-015-0750-z) contains supplementary material, which is available to authorized users.

## Background

Malaria is among the world’s most devastating and widespread infectious diseases and represents a significant barrier to socio-economic development in the world’s poorest countries [1–3]. The deadliest form of malaria is caused by the parasite *Plasmodium falciparum* and the clinical symptoms are associated with the blood stages of infection when merozoite-stage parasites invade, multiply within and then destroy host erythrocytes. The most severe outcome is cerebral malaria that leads to high mortality and can cause permanent brain injury to surviving patients. *Plasmodium* parasites have evolved effective immunoregulatory mechanisms so that sterile immunity develops very slowly, if at all, and is rapidly lost in areas of lower transmission [4]. Consequently, there is a pressing need for novel therapeutics, particularly a vaccine, the rational design of which will be greatly aided by a deeper understanding of the molecular mechanisms by which host and pathogen interact.

Extracellular host and pathogen factors are at the nexus of host-parasite interactions and often mechanistically explain and determine the pathological outcome of infection. P-selectin (SELP, also known as CD62P) is a host cell surface receptor protein that is known to influence malaria-associated pathology, both in human patients and rodent infection models [5–8]. P-selectin is required for the efficient recruitment of circulating leukocytes to sites of localized inflammation, being rapidly up-regulated on the surface of inflamed vascular endothelial cells [9, 10]. P-selectin is a lectin which binds the Sialyl-Lewis^X^ (SLe^X^) tetrasaccharides displayed on leukocyte cell surface glycoproteins, particularly PSGL-1 (P-Selectin Glycoprotein Ligand 1) [11]. Patients deficient in P-selectin function suffer from persistent bacterial infections, demonstrating the importance of this protein in regulating the response to infection in humans [12]. There is also increasing evidence that P-selectin plays an important role in *Plasmodium* infections. In humans, there is evidence that increased levels of a secreted form of P-selectin are found in patients presenting with non-severe *versus* severe malaria [5], possibly by out-competing and reducing interactions between membrane-tethered endothelial P-selectin and the parasite ligand PfEMP1, which is displayed on the surface of infected erythrocytes [8, 13]. Consistent with this, P-selectin accumulates in the brains of mouse strains that are susceptible to experimental cerebral malaria (ECM), but not in resistant strains [6]; also, ECM-susceptible mouse strains that are P-selectin-deficient do not succumb to cerebral pathology [6]. Together, these data suggest that P-selectin-mediated inflammatory responses contribute to the pathology of severe cerebral malaria; however, because blood cell sequestration does not appear to be altered in the brains of P-selectin-deficient mice, the mechanism by which P-selectin appears to exacerbate severe disease is still unexplained and could be distinct from its known adhesive roles [7].

A systematic protein interaction screening method (AVEXIS, for AVidity-based EXtracellular Interaction Screen) that is specifically designed to identify extracellular protein interactions was used to further investigate the mechanistic basis of P-selectin function in the pathogenesis of malaria. Using a highly avid P-selectin binding regent and a panel of *P. falciparum* merozoite proteins, a direct interaction between P-selectin and merozoite surface protein 7 (MSP7) was identified. *Plasmodium falciparum* MSP7 forms part of the abundant MSP1 complex on the surface of merozoites and the phenotypes of MSP7-deficient parasites suggest that this protein plays an important, but non-essential, role in erythrocyte invasion [14–16].

In this work, the interaction between P-selectin and PfMSP7 was characterized biochemically and it was shown that P-selectin function was impaired in the presence of PfMSP7 because it could no longer interact with its endogenous glycan ligand Sialyl-Lewis^X^. Interactions between P-selectin and the wider MSP7 protein family could, therefore, play an immunomodulatory role, separate from PfMSP7’s role in erythrocyte invasion, during *Plasmodium* infections.

## Methods

### Expression and purification of recombinant proteins

The plasmids encoding the entire ectodomains of 17 *P. falciparum* merozoite proteins and human P-selectin were described previously [17–19]; the ectodomain of mouse P-selectin was amplified from a plasmid encoding a full-length cDNA (Origene, NM_011347.1). Regions encoding sub-regions or defined domains of PfMSP7 and P-selectin were amplified by PCR from plasmids encoding the entire ectodomain and cloned into appropriate expression plasmids. Boundaries for each of the recombinant P-selectin protein fragments were as follows: M_1_ANC…IQEA_771_ (1), M_1_ANC…CEAI_570_ (2), M_1_ANC…EAIS_385_ (3), M_1_ANC…EYVR_208_ (4), M_1_ANC…YTAS_162_ (5), Y_159_TAS…IQEA_208_ (6), where amino acid numbers pertain to P16109 in Uniprot and correspond to those in Fig. 4a. Similarly, the PfMSP7 fragments shown in Fig. 4b are delineated by the following boundaries: T_1_PVN…LNTM_351_ (PfMSP7), T_1_PVN…VKAQ_176_ (PfMSP7-N), S_177_ETD…LNTM_351_ (PfMSP7_22_), E_195_VQK…LNTM_351_ (PfMSP7_19_) where amino acid numbers correspond to the PF3D7_1335100 protein sequence.

The ectodomains were expressed as soluble recombinant proteins by transient transfection of HEK293E or HEK293F cells with a C-terminal rat Cd4 domains 3 and 4 tag and with either a biotinylated C-terminal tag for immobilization on streptavidin or a 6-His tag for purification [19] and processed as described [20]. Where appropriate, proteins were purified from transfected-HEK293E cell culture supernatants on HisTrap HP columns using an ÄKTAxpress instrument (GE Healthcare) as described [21].

### AVidity-based EXtracellular protein Interaction Screening (AVEXIS)

AVEXIS assays were performed essentially as described previously [20, 22]. Briefly, biotinylated ‘bait’ proteins were immobilized in a well of a streptavidin-coated, 96-well, microtitre plate at a concentration that saturated the biotin binding capacity of the well. After a brief wash, the bait arrays were probed with a highly avid (pentamerized) β-lactamase-tagged ‘prey’ normalized to previously determined activity levels [20, 22]. Prey capture was quantified by measuring the absorbance of the hydrolysis product of nitrocefin – a colorimetric β-lactamase substrate – at 485 nm after one hour. Typically, a mean background absorbance value was calculated from negative controls, and subtracted from the absorbance in each query well.

Where the anti-P-selectin antibody was used in blocking experiments, immobilized P-selectin bait proteins were first incubated with a range of concentrations of the CLB-thromb/6 monoclonal antibody (Santa Cruz Biotechnology Inc) for one hour, prior to incubation with the PfMSP7 prey. Where appropriate, both the full-length P-selectin bait and PfMSP7 prey proteins were pre-incubated with a range of EDTA concentrations (maximum 10 mM) for one hour before use in AVEXIS.

### Flow cytometry

Human P-selectin was expressed on the surface of transfected cells with its cytoplasmic region replaced by a fluorescent marker by sub-cloning the entire P-selectin ectodomain into a plasmid containing a transmembrane domain and cytoplasmic eGFP, as described [23]. HEK293F cells were transiently transfected with this plasmid and 24 hours later, ~1×10^6^ cells were incubated with 5 μg of purified FLAG-tagged pentameric prey proteins for one hour at 4 °C. Cells were washed with PBS and stained with 2 μg Cy3-conjugated anti-FLAG M2 monoclonal antibody (Sigma-Aldrich). Cells were washed and resuspended in 1 mL PBS and analysed on a Becton-Dickinson LSR Fortessa flow cytometer. Where appropriate, cells were pre-incubated with 10 μg mouse monoclonal IgG1 antibodies for one hour at 4 °C prior to addition of the FLAG-tagged prey. These antibodies included the CLB-thromb/6 anti-P-selectin clone and the OX102 anti-rat Cd200R clone (BioLegend).

### Surface plasmon resonance

Surface plasmon resonance studies were performed using a Biacore T100 instrument (GE Healthcare) at a temperature of 37 °C in HBS buffer. Approximately 150 RU of a negative control bait (biotinylated rat Cd4 domains 3 + 4) was immobilized in a streptavidin-coated sensor chip in a reference flow cell and the binding responses subtracted from those in the query flow cell in which an approximately molar equivalent of P-selectin was immobilized. Purified PfMSP7 was buffer exchanged in HBS and resolved by size exclusion chromatography using a Superdex 2000 Increase 10/300 column (GE Healthcare) prior to Biacore analysis. PfMSP7 formed metastable oligomeric forms, the relative amounts of which were preparation-dependent and did not correlate simply with either protein concentration or storage time. Increasing concentrations of purified PfMSP7 analyte were injected over both flow cells at a flow rate of 100 μl/min and the chip surface regenerated at the end of each cycle. Owing to the strength of this interaction, regeneration of the chip surface required repeated injections of 5 M NaCl. Responses of duplicate concentrations gave the same binding signals after a regeneration cycle, demonstrating that the regeneration solution did not result in any loss of activity of the protein immobilized on the chip.

### P-selectin-Sialyl Lewis^X^ binding assays

A biotinylated derivative of SLe^X^ (Glycotech) was clustered around a streptavidin-alkaline phosphatase conjugate (Sigma) to create an avid enzyme-tagged SLe^X^ binding reagent (SLe^X^-AP). A 5:1 ratio of SLe^X^ to streptavidin-AP was found to be optimal to maximize the P-selectin binding response. Monobiotinylated P-selectin was immobilized on a streptavidin-coated microtitre plate and probed with a dilution series of SLe^X^-AP, incubated for one hour at room temperature (approximately 22 °C). Plates were washed three times with HBS containing 0.2 % Tween20 and then 100 μL of 1 mg/mL phosphatase substrate (4-Nitrophenyl phosphate disodium salt) was added to each well. SLe^X^-AP binding was quantified by measuring the absorbance of the hydrolysis products at 405 nm, 30 minutes after substrate addition. To determine whether PfMSP7 could block the interaction, immobilized P-selectin was incubated with PfMSP7 pentamers for one hour prior to washing before addition of 100 μL 0.1nM SLe^X^-AP.

## Results

### A systematic extracellular protein interaction screen identified P-selectin as a receptor for PfMSP7

**Fig. 1:**
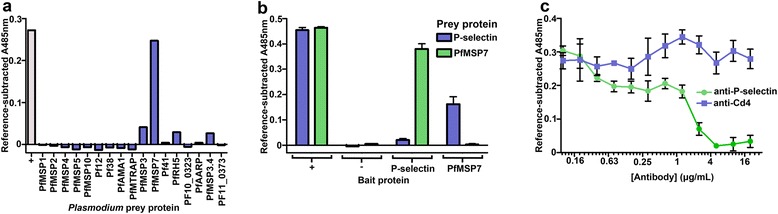
Systematic extracellular protein interaction screening by AVEXIS identified PfMSP7 as a ligand for human P-selectin. **a** The entire ectodomains of the 17 named *P. falciparum* merozoite proteins were screened as highly avid β-lactamase-tagged prey proteins against P-selectin bait using the AVEXIS method. Prey binding was quantified by β-lactamase hydrolysis of a colorimetric substrate at 485 nm. P-selectin bound clearly and specifically to PfMSP7. Positive (+) represents mean value for total capture of all preys with an anti-prey bait antibody. **b** PfMSP7 and P-selectin prey proteins were screened against PfMSP7 and P-selectin bait proteins as well as an OX68 positive control bait (+) and a rat Cd4 negative control bait (−) by AVEXIS. Interactions were detected in both bait:prey orientations. **c** An anti-P-selectin mAb blocked the interaction between P-selectin bait and PfMSP7 prey in a dose-dependent manner relative to an anti-Cd4 mAb control, which bound to the tag region of the P-selectin protein, in an AVEXIS assay. Bars in (**b**) and (**c**) represent means ± SD, *n* = 3. Representative experiment of more than three independent experiments are shown

To identify potential *Plasmodium* ligands for P-selectin, the entire ectodomains of 17 *P. falciparum* merozoite ligands [17] were screened for interactions against the entire ectodomain of human P-selectin using AVEXIS. AVEXIS is a systematic approach specifically developed to identify low affinity extracellular interactions and works by detecting direct binding events within libraries of recombinant proteins expressed by mammalian cells [22]. Briefly, ‘bait’ proteins, in this case P-selectin and controls, are arrayed in microtitre plates and probed with enzyme-tagged ‘preys’, in this case merozoite ligands, which have been purposefully clustered into pentamers thereby increasing overall binding avidity to detect even the most fleeting of interactions. Reproducible and robust binding signals were observed using the PfMSP7 prey with the P-selectin bait (Fig. 1a). One simple and informative validation criteria for interactions detected using the AVEXIS approach is to ask whether the interaction is dependent on the bait-prey orientation [22]. To test this, P-selectin was produced as a β-lactamase-tagged prey and presented to both immobilized PfMSP7 and control baits. Again, clear binding was observed, although with a reproducibly lower signal, possibly due to reduced prey activity caused by homophilic P-selectin interactions between prey molecules (Fig. 1b); this prey ‘masking’ effect due to homophilic interactions has been observed previously [22].

PfMSP7 is proteolytically processed and a C-terminal fragment associates with and forms part of the MSP1 protein complex located on the merozoite surface [16, 24]. To verify that the recombinant PfMSP7 prey protein preparation was biologically active, its ability to bind the PfMSP1 bait protein was demonstrated using the AVEXIS assay as previously reported (panel a in Additional file 1: Figure S1) [17]. Similarly, the recombinant P-selectin prey bound to a SLe^X^ bait (panel a in Additional file 1: Figure S1) and a P-selectin bait bound to an avid SLe^X^-alkaline phosphatase conjugate (panel b in Additional file 1: Figure S1), again confirming the biological activity of the recombinant P-selectin protein. To further confirm the specificity of the interaction, a monoclonal antibody to P-selectin was used to block the interaction in a dose-dependent manner (Fig. 1c). The epitope of the anti-P-selectin monoclonal antibody CLB-thromb/6, which blocked the P-selectin-MSP7 interaction, is located between the N-terminal C-type lectin domain and EGF-like domain of P-selectin [25]. Because the SLe^X^ binding properties of P-selectin’s C-type lectin domain are dependent on calcium ions, the necessity of calcium for the interaction with PfMSP7 was investigated. A concentration series of the divalent cation chelator EDTA was used to demonstrate that approximately 30 mM EDTA was sufficient to prevent the interaction from occurring (panel c in Additional file 1: Figure S1), which is in good agreement with the concentration that was previously used to block other P-selectin-binding interactions [26]. Together, these data identify PfMSP7 as a possible ligand for P-selectin.

### PfMSP7 specifically binds P-selectin at the surface of cells

**Fig. 2:**
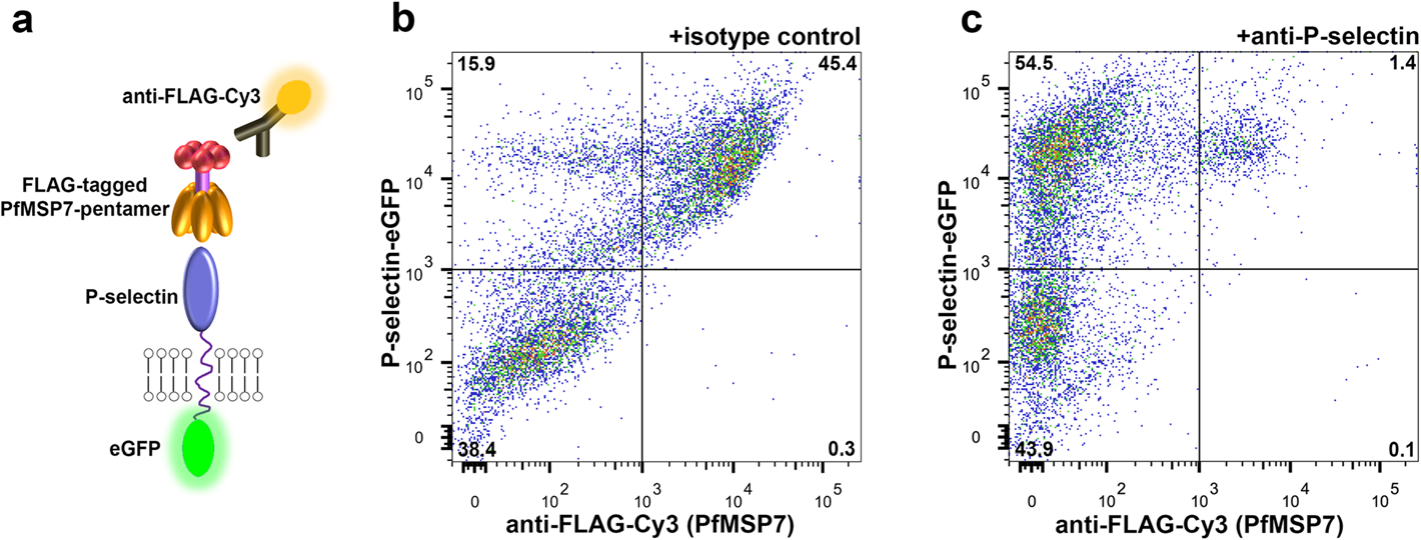
PfMSP7 interacted with P-selectin at the surface of cells. **a** Schematic representing the experimental design, whereby recombinant, pentameric PfMSP7 proteins interact with eGFP-tagged P-selectin at the surface of transfected cells. **b** The interaction of P-selectin-eGFP transfected cells with FLAG-tagged pentameric PfMSP7 was detected by flow cytometry. Pre-incubation of cells with anti-Cd200R negative control mAb did not impair interaction detection. **c** P-selectin-GFP transfected cells incubated with CLB-thromb/6 anti P-selectin mAb prior to incubation with FLAG-tagged pentameric PfMSP7 could not replicate this interaction observed in (**b**)

While the use of recombinant proteins in reductionist *in vitro* assays allows important experimental control over binding parameters, it does not satisfactorily replicate the complex environment of cell surfaces in which the interaction would normally occur. To replicate the elements of this environment, a P-selectin expression plasmid was constructed in which the cytoplasmic region was replaced with an eGFP reporter protein and then displayed at the surface of human cells by transiently transfecting the HEK293F cell line. Cells transfected with this construct were incubated with a pentamerized FLAG-tagged PfMSP7 prey protein and binding quantitated with an anti-FLAG fluorescent secondary antibody using flow cytometry relative to a control prey (Fig. 2a). A clear double-positive population was observed demonstrating that P-selectin-expressing (GFP-positive) cells interacted with the PfMSP7 FLAG-tagged prey (Fig. 2b). Within the sample of transfected cells used in each staining reaction, the level of PfMSP7 binding was proportional to the P-selectin-GFP expression level, and, importantly, the untransfected (GFP-negative) cells within the population did not bind PfMSP7 (Fig. 2b). The P-selectin dependency of PfMSP7 binding was further established by preventing binding by first pre-incubating the transfected cells with the anti-P-selectin antibody (Fig. 2c). These data provide additional evidence that MSP7 and P-selectin specifically interact.

### PfMSP7 forms multimers and directly interacts with P-selectin with high avidity

**Fig. 3:**
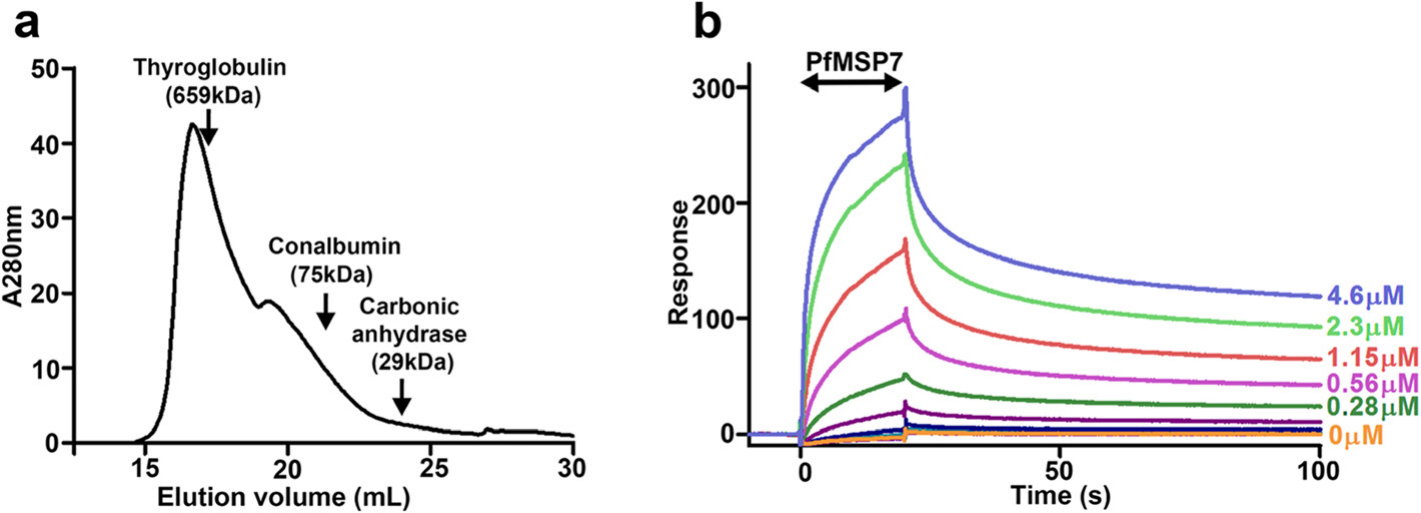
Full length PfMSP7 forms multimers and directly interacts with P-selectin with high avidity. **a** Purified recombinant full-length PfMSP7 was resolved by size-exclusion chromatography and eluted as a polydisperse peak. This was typical of more than ten independent preparations, though some elution profiles showed additional peaks at higher elution volumes. **b** Analysis of the PfMSP7/P-selectin interaction by SPR. A dilution series of the major peak of resolved PfMSP7 was sequentially injected over P-selectin immobilized on a streptavidin-coated sensor chip. SPR responses upon 20 s injections of the indicated concentrations of purified PfMSP7 analyte over P-selectin bait

To demonstrate that P-selectin and PfMSP7 interact directly and to determine the biophysical binding parameters of the interaction, surface plasmon resonance (SPR) was used. The full length PfMSP7 protein was expressed as a recombinant protein, purified and analysed by size exclusion chromatography (SEC). Multiple preparations of purified PfMSP7 protein repeatedly resolved as a heterogenous polydisperse mixture of species, typically containing a single predominant peak consistent with the formation of higher-order oligomers, but with varying amounts of slower-eluting smaller forms of lower molecular mass, which were preparation-dependent, suggesting that full length PfMSP7 forms metastable oligomers in solution (Fig. 3a). Multimeric assemblies of parasite proteins are known to be important for their critical interactions with host proteins [27], and similar oligomerization behaviour to that reported here has been observed for the *Plasmodium* surface proteins PfMSP2 and PfMSP3 [28–31].

After resolution by SEC, serial dilutions of the main species of purified PfMSP7 were injected over P-selectin bait immobilized on the surface of an SPR chip. Clear binding responses were observed, demonstrating that the two proteins interacted directly (Fig. 3b). As expected, given the oligomeric state of PfMSP7, the shape of the binding curves indicated complex multivalent binding behaviour which did not fit well to a simple 1:1 binding model (Fig. 3b). A rapid initial binding was followed by much slower association, which did not achieve equilibrium over the course of the injection phase which precluded the calculation of an equilibrium dissociation constant. The slows dissociation of the two proteins during the wash-out phase shows that the binding is highly avid, which is typical of a higher affinity interaction between a secreted protein and a membrane-tethered receptor.

### PfMSP7’s interaction site is located within the SLe^X^-binding C-type lectin and EGF domains of P-selectin

**Fig. 4:**
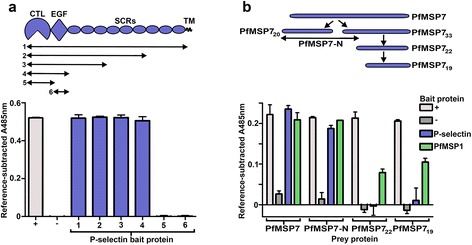
The PfMSP7 interaction site is located within the C-type lectin and EGF domains of P-selectin. **a** A schematic showing the extent of the six P-selectin bait truncations that were expressed and then screened for binding to a full-length PfMSP7 prey using the AVEXIS assay. The shortest regions that retained PfMSP7 binding encompassed both the CTL and EGF domains. **b** P-selectin bait was screened against PfMSP7 fragment prey proteins by AVEXIS. Schematic shows the regions of the PfMSP7 proteins used in this assay, alongside the scheme of PfMSP7 processing described in the text. Bars represent mean + SD *n* = 3. Representative experiments of two independent experiments are shown. In each experiment the positive controls (+) were PfMSP7 preys screened against OX68 bait and negative controls (−) were PfMSP7 preys screened against the Cd4 tag bait

To further characterize the interaction, the regions on both P-selectin and PfMSP7 responsible for binding were identified. P-selectin consists of an N-terminal C-type lectin (CTL) domain that forms the calcium-dependent SLe^X^ binding site followed by an epidermal growth factor (EGF) domain, and nine short consensus repeat (SCR) domains (Fig. 4a). A panel of structure-guided C-terminally-truncated P-selectin bait proteins was expressed and screened against a PfMSP7 prey using the AVEXIS assay (Fig. 4a). A fragment consisting of just the N-terminal CTL and EGF domains bound PfMSP7 indistinguishably from the entire P-selectin ectodomain, demonstrating that the PfMSP7 binding site was located in the SLe^X^-binding N-terminal region of P-selectin. To further resolve the binding region to either the CTL or EGF domains, both domains were expressed individually; however, both domains were minimally required to interact with full-length PfMSP7 suggesting that the binding site spans both domains, or there is a structural requirement that both domains are functionally expressed as a discrete unit (Fig. 4a).

PfMSP7 is proteolytically processed within the merozoite, being initially cleaved into two major forms: a 20 kDa N-terminal fragment that can be detected within the parasitophorous vacuole [32], and a larger C-terminal fragment which is further processed to leave a 22 kDa fragment (PfMSP7_22_) that can form part of the MSP1 complex, a major component of the merozoite surface. In most strains of *P. falciparum*, PfMSP7_22_ can be cleaved again at the surface of the merozoite to leave PfMSP7_19_ in the MSP1 complex [24]. To determine which part of PfMSP7 bound P-selectin, prey proteins corresponding to PfMSP7_19_, PfMSP7_22_ and the N-terminal 20 kDa region (referred to hereafter as PfMSP7-N) were expressed and tested for binding to P-selectin by AVEXIS. PfMSP7-N - the portion that is not part of the MSP1 complex on the merozoite surface - interacted robustly with P-selectin; the PfMSP7_19_ and PfMSP7_22_ prey did not interact (Fig. 4b). This is a particularly interesting result, as no function has previously been identified for the N-terminal fragments of the PfMSP7 precursor protein. As expected, the PfMSP7_19_ and PfMSP7_22_ prey interacted with PfMSP1, as did PfMSP7-N, which is consistent with the finding that the immature PfMSP1 and PfMSP7 proteins associate prior to processing [24], and also suggests that there may be multiple binding sites for PfMSP7 on PfMSP1.

### PfMSP7 pentamers block the interaction between P-selectin and SLe^X^

**Fig. 5:**
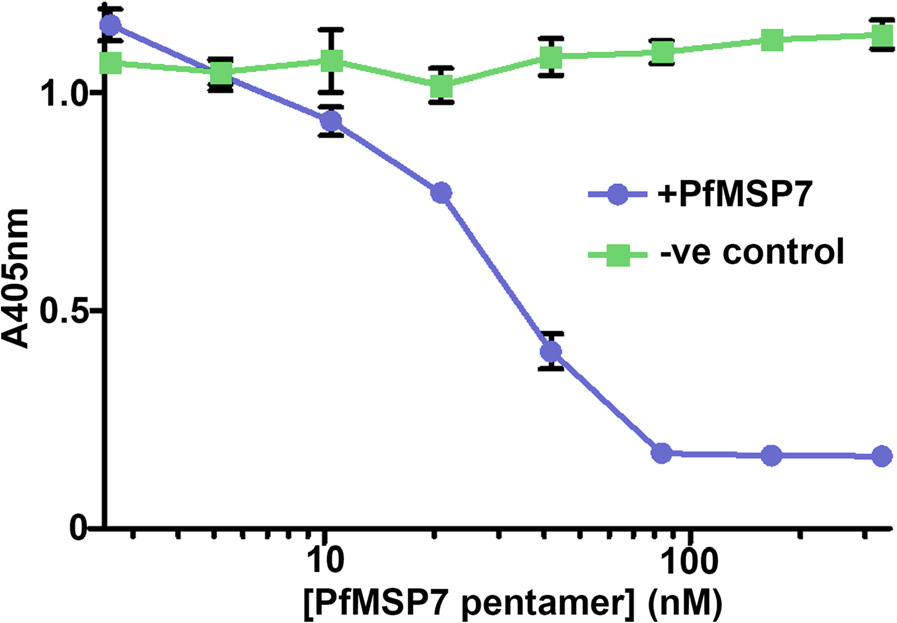
PfMSP7 can block the interaction between P-selectin and its glycan ligand Sialyl-Lewis^X^. P-selectin bait was immobilized and pre-incubated with the indicated concentrations of the PfMSP7 pentamer before addition of an avid alkaline-phosphatase-conjugated SLe^X^ binding reagent. PfMSP7 pentamers blocked SLe^X^ binding to immobilized P-selectin. Data points represent mean ± SD, *n* = 3; a representative experiment from two independent experiments is shown

The finding that PfMSP7 interacted with the CTL domain of P-selectin suggested the possibility that it could affect the binding of its endogenous ligand, SLe^X^, and thereby prevent the recruitment of leukocytes to inflamed endothelium. To determine whether PfMSP7 could block the interaction between P-selectin and SLe^X^, a dilution series of purified pentameric PfMSP7 was added to the P-selectin-SLe^X^ binding assay used previously (panel b in Additional file 1: Figure S1). This showed that PfMSP7 could inhibit SLe^X^ binding in a concentration-dependent manner (Fig. 5). These data, combined with the information from the domain mapping on P-selectin, demonstrate that PfMSP7 can compete with SLe^X^ -containing ligands for P-selectin binding, and could therefore disrupt the normal pro-adhesive and pro-inflammatory functions of P-selectin.

### The MSP7-P-selectin interaction is conserved within paralogues and between orthologues

**Fig. 6:**
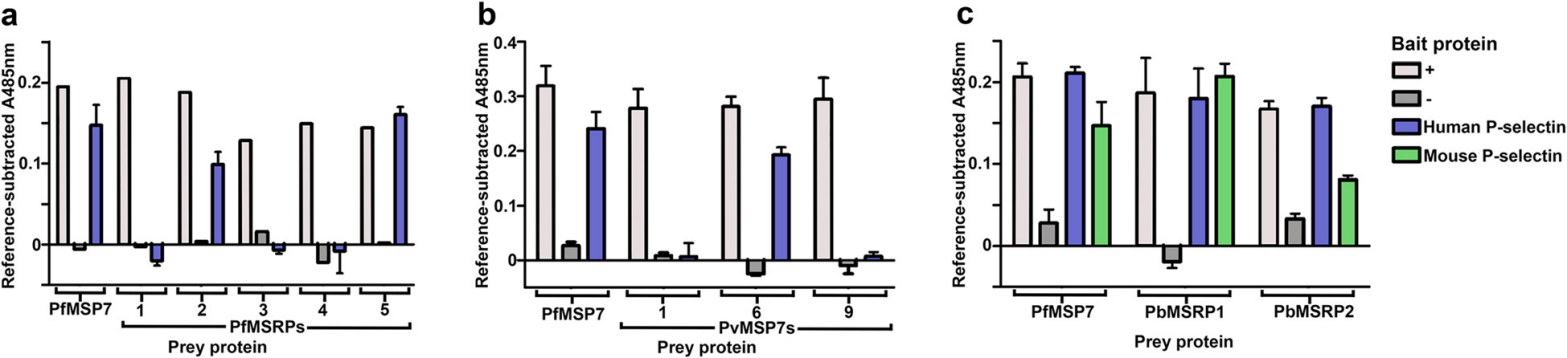
P-selectin binding is conserved within the wider MSP7 family across different *Plasmodium* species. P-selectin-MSP7 binding assays using AVEXIS were used to determine the conservation of binding within the MSP7 orthologues and paralogues of *P. falciparum* (**a**), *P. vivax* (**b**), and *P. berghei* (**c**). **a** P-selectin bound to PfMSRP2 and 5, but not PfMSRP1, 3 or 4. **b** Detectable P-selectin binding was observed with PvMSP7-6, but not PvMSP7-1 or −9. *P. vivax* MSP7s are labelled according to their arrangement on chromosome: 1 = PVX_082700, 6 = PVX_082675, and 9 = PVX_082655. **c** Both PbMSRP1 and 2 bound both human and mouse P-selectin baits. Bars represent mean + SD, *n* =3. A representative experiment from at least two independent experiments is shown in each case. In each experiment the positive controls (+) were PfMSP7 preys screened against OX68 bait and negative controls (−) were PfMSP7 preys screened against the Cd4 tag bait

MSP7 is part of a paralogous gene family found in all sequenced *Plasmodium* spp. genomes. Pf*MSP7* and at least five *MSP7*-related genes are located in tandem arrays on chromosome 12 of *P. falciparum* [32, 33]. These genes have a similar structure and organization, indicating that members of this family have arisen through gene duplication, although there is very little protein sequence conservation between their N-termini. To assess whether P-selectin binding is a conserved function within PfMSP7 paralogues, the full length sequences of all five MSP7-related proteins (MSRPs) from *P. falciparum* were expressed as prey proteins and tested for their ability to bind P-selectin bait by AVEXIS. Of the five proteins, binding was detected between P-selectin and both PfMSRP2 and PfMSRP5 (Fig. 6a). To determine whether the interaction is conserved between other *Plasmodium* species that infect humans, three of the approximately 11 MSP7-like proteins encoded within the *Plasmodium vivax* genome were expressed as prey proteins. Of these, clear binding was observed with PVX_082675 (PvMSP7-6) (Fig. 6b). Finally, recombinant *Plasmodium berghei* MSP7s were used to determine whether this potential immunomodulatory interaction was a conserved feature of *Plasmodium* infections in other mammalian species. Prey plasmids for all three MSP7-like genes from the rodent parasite *Plasmodium berghei* were designed, and although full length proteins for both PbMSRP1 and PbMSRP2 were expressed, repeated attempts failed to yield sufficient protein for PbMSP7. Again using AVEXIS, binding was detected between PbMSRP1 and PbMSRP2 and the mouse P-selectin orthologue (Fig. 6c). The conservation of binding to P-selectin within the wider MSP7 family across different *Plasmodium* species suggests that this interaction plays an important immunoregulatory role during *Plasmodium* infections of mammalian hosts.

## Discussion

Here, the identification and characterization of interactions occurring between human P-selectin and *Plasmodium* MSP7 proteins are described for the first time. Prior to this work, much of the existing knowledge about PfMSP7 pointed to a role in erythrocyte invasion based on the original observation that a processed C-terminal fragment of MSP7 co-purified and directly interacted with the major merozoite surface protein, MSP1 [16, 24]. Consistent with this, *PfMSP7*-deficient parasites exhibited subtle (~30 %) reductions in the ability of the parasites to invade human erythrocytes *in vitro* [15], and antibodies raised against PfMSP7 have also been shown to impair invasion [34]. Similarly, *MSP7*-deficient *P. berghei* parasites are growth-attenuated compared to their wild-type counterparts in rodent models of infection [14, 35–37]. Interestingly, a proteomic analysis of *P. falciparum* blood-stage parasites selected to invade erythrocytes independently of host sialic acid demonstrated a significant up-regulation of PfMSP7, suggesting varying levels of PfMSP7 may modulate erythrocyte recognition [38].

However, it is likely that MSP7 proteins also perform additional roles that do not necessarily relate to erythrocyte invasion. Multiple MSP7 proteins are transcribed in *P. falciparum* parasites, yet there is little evidence to suggest that any of the MSRP proteins can substitute for PfMSP7’s role in the MSP1 complex [39]. Evidence from rodent infections with ΔPbMSP7 parasites indicates that PbMSP7 may have an immunomodulatory function; ΔPbMSP7 parasites, compared to their wild-type counterparts, were substantially less deadly to their hosts, and ΔPbMSP7 parasites induced much less cerebral pathology in ECM-susceptible mice, in a manner that could not be entirely attributed to reduced parasitaemia or anaemia [35, 36]. Differences in splenic clearance and induction of CD8+ T cell population expansion are also observed between wild-type and ΔPbMSP7 parasite infections [37].

The discovery that the N-terminal region of PfMSP7 is a ligand for the host receptor P-selectin - a protein that has well documented roles in leukocyte recruitment to sites of inflamed endothelium - suggests a new functional role for PfMSP7 that is distinct from invasion. This conclusion is supported both by the absence of P-selectin from human erythrocytes [40] and because the N-terminal region of PfMSP7, which has been shown here to interact with P-selectin, has not been detected on the merozoite surface, indicating that the different fragments of PfMSP7 have separate biological roles. The suggestion that the role of MSP7-family proteins is not limited to promoting erythrocyte invasion is further supported by evidence that many MSP7-family proteins are secreted, rather than being localized at the merozoite surface; for example, both PfMSRP5 [33] and the N-terminus of PfMSP7 [32], when transgenically fused with GFP, are located within the parasitophorous vacuole (PV), and the solubility of PfMSRP2 in cultured parasites suggests that it is also exported from the parasite into the PV [39]. It is therefore likely that MSP7-family proteins and their processed fragments are released into the host bloodstream upon schizont rupture, so that they are able to directly interact with host proteins free in the blood or those displayed on blood cells or vascular endothelium.

Having taken the view that MSP7-P-selectin interactions are unlikely to be involved in erythrocyte invasion, a model can be suggested whereby MSP7 binding to P-selectin acts to limit inflammatory processes and therefore impacts parasite, and possibly host, survival. Such a role could help to provide a molecular basis for the reported delay in death in ΔPbMSP7 parasitized, ECM-resistant mice, compared to those infected with wild-type parasites; by deploying MSP7 proteins to block the interaction between leukocytes and the endothelium, *Plasmodium* parasites could potentially dampen the production of pro-inflammatory cytokines and phagocytic activity by leukocytes [41], thus aiding parasite survival. The *in vitro* evidence that PfMSP7 is capable of blocking the interaction P-selectin makes with leukocyte ligands indicates that this is theoretically possible, although *in vivo* it would undoubtedly be part of a much more complex set of immunomodulatory mechanisms employed by the *Plasmodium* parasite.

Given the complementary evidence that P-selectin-deficient mice do not develop cerebral malaria [6] and that ECM pathology is limited in susceptible mice infected with ΔPbMSP7 parasites [36], it is tempting to speculate that the interaction between P-selectin and MSP7 exacerbates the pathological processes involved in the development of cerebral malaria. This could conceivably be an indirect consequence of MSP7 proteins interacting with soluble as opposed to membrane-bound P-selectin, impairing the ability of free P-selectin to limit inflammatory interactions and thus promoting systemic inflammation.

## Conclusion

These results demonstrate a novel interaction between a human cell surface protein and a family of *Plasmodium* proteins; since this interaction appears to have been evolutionarily conserved in a range of parasite species, this interaction may be beneficial to the parasite, allowing it to modulate the host’s response to infection. If an impairment of the capacity of MSP7 proteins to bind P-selectin is found to underlie the defects in immune evasion and lethality seen in ΔMSP7 *P. berghei* parasites, then the potential to block this interaction from occurring *in vivo* provides a new target for therapeutic interventions that could mitigate illness and death from malaria.

## Additional file

Additional file 1: Figure S1. Recombinant P-selectin and PfMSP7 were functionally active, and their interaction was EDTA-sensitive. **a** Recombinant P-selectin and PfMSP7 were screened against known receptors, SLe^X^ and PfMSP1 respectively by AVEXIS. The P-selectin prey bound to SLe^X^ bait whilst the PfMSP7 prey bound to PfMSP1 demonstrating that the recombinant prey proteins are able to perform the normal binding functions of their native counterparts. Both prey bound to an anti-tag (OX68) positive control antibody bait (+) and neither prey bound to the negative control Cd4 tag bait (−). **b** Immobilized P-selectin bait protein bound SLe^X^-alkaline phosphatase (AP) conjugate, demonstrating that the bait protein, like the P-selectin prey in (**a**), is able to perform the binding functions of the active native protein. P-selectin was unable to bind to the unconjugated streptavidin-AP used as a negative control (−). **c** P-selectin/PfMSP7 interaction detection by AVEXIS was sensitive to divalent cation chelation by EDTA, which prevented the interaction in a dose-dependent manner. The control interaction between rat Cd200 and Cd200R was not affected by the presence of EDTA. Error bars represent the mean absorbance values +/− SD, *n* = 3.
