# Supplementary figures and images for: P-selectin is a host receptor for Plasmodium MSP7 ligands
Source: Malar J. 2015 Jun 5;14:238. doi: 10.1186/s12936-015-0750-z (PMC4478713; doi:10.1186/s12936-015-0750-z)

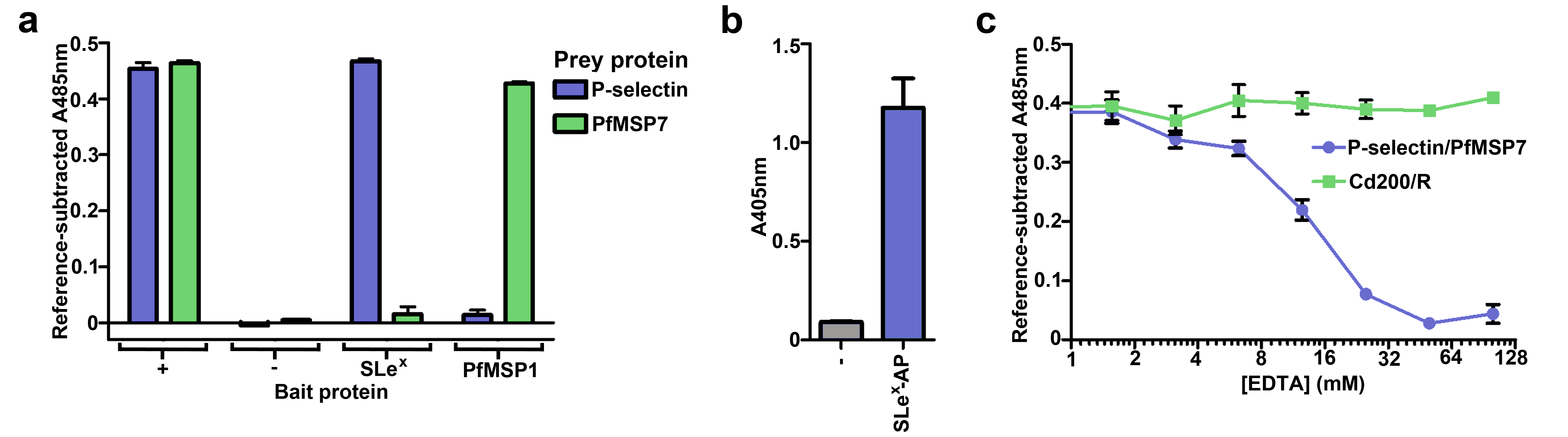

Supplement: Additional file 1: Figure S1. — Recombinant P-selectin and PfMSP7 were functionally active, and their interaction was EDTA-sensitive. a Recombinant P-selectin and PfMSP7 were screened against known receptors, SLeX and PfMSP1 respectively by AVEXIS. The P-selectin prey bound to SLeX bait whilst the PfMSP7 prey bound to PfMSP1 demonstrating that the recombinant prey proteins are able to perform the normal binding functions of their native counterparts. Both prey bound to an anti-tag (OX68) positive control antibody bait (+) and neither prey bound to the negative control Cd4 tag bait (−). b Immobilized P-selectin bait protein bound SLeX-alkaline phosphatase (AP) conjugate, demonstrating that the bait protein, like the P-selectin prey in (a), is able to perform the binding functions of the active native protein. P-selectin was unable to bind to the unconjugated streptavidin-AP used as a negative control (−). c P-selectin/PfMSP7 interaction detection by AVEXIS was sensitive to divalent cation chelation by EDTA, which prevented the interaction in a dose-dependent manner. The control interaction between rat Cd200 and Cd200R was not affected by the presence of EDTA. Error bars represent the mean absorbance values +/− SD, n = 3. [file 12936_2015_750_MOESM1_ESM.tif]
